# Supplementary material for: CRISPR Cas13-Based Tools to Track and Manipulate Endogenous Telomeric Repeat-Containing RNAs in Live Cells
Source: Front Mol Biosci. 2022 Jan 31;8:785160. doi: 10.3389/fmolb.2021.785160 (PMC8841788; doi:10.3389/fmolb.2021.785160)
Supplement: Supplementary file 1 [file Table1.docx]

Supplemental Table 1. Anomalous exponent and mean diffusion coefficient in Figure 3D and Figure S1C.

|  | **Time scale: 0-1 second** | | **Time scale: 10-100 second** | |
| --- | --- | --- | --- | --- |
|  | **Non-Telomeric** | **Telomeric** | **Non-Telomeric** | **Telomeric** |
| **Mean Anomalos Exponent (α)** | 0.52 ± 0.04 | 0.34 ± 0.19 | 0.70 ± 0.33 | 0.64 ± 0.33 |
| **Mean Diffusion Coefficient (µm^2^/s)** | 0.037 ± 0.020 | 0.013 ± 0.011 | 0.0033 ± 0.0007 | 0.0029 ± 0.001 |
