# Supplementary figures and images for: CRISPR Cas13-Based Tools to Track and Manipulate Endogenous Telomeric Repeat-Containing RNAs in Live Cells
Source: Front Mol Biosci. 2022 Jan 31;8:785160. doi: 10.3389/fmolb.2021.785160 (PMC8841788; doi:10.3389/fmolb.2021.785160)

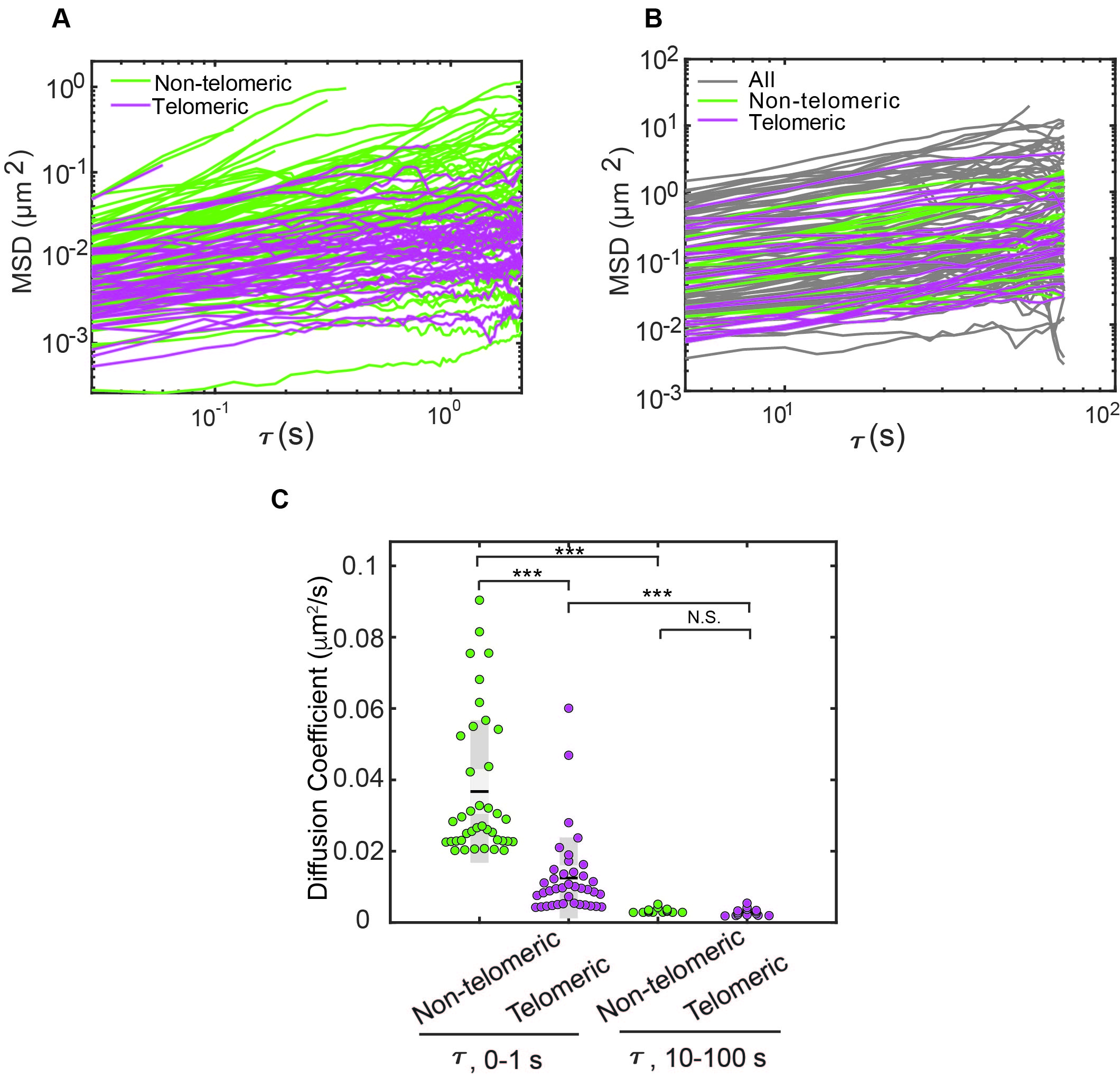

Supplement: Supplementary file 2 [file Image1.jpeg]
